# NK cells limit therapeutic vaccine-induced CD8^+^T-Cell immunity in a PDL1-dependent manner

**DOI:** 10.1126/scitranslmed.abi4670

**Published:** 2022-04-13

**Authors:** Mariana O. Diniz, Anna Schurich, Senthil K. Chinnakannan, Marion Duriez, Kerstin A. Stegmann, Jessica Davies, Stephanie Kucykowicz, Kornelija Suveizdyte, Oliver E. Amin, Frances Alcock, Tamsin Cargill, Eleanor Barnes, Mala K. Maini

**Affiliations:** 1 Division of Infection and Immunity and Institute of Immunity and Transplantation, UCL, London, UK; 2 Peter Medawar Building for Pathogen Research, Nuffield Dept of Medicine, University of Oxford, Oxford, UK

## Abstract

A better understanding of regulatory mechanisms limiting the CD8^+^T-cell response to therapeutic vaccines is urgently needed in order to develop adjunctive approaches to enhance their efficacy in chronic viral infections and cancers. We show that NK cell depletion enhances antigen-specific T cell responses to chimp adenoviral vector (ChAdOx) vaccination in a mouse model of chronic HBV infection (CHB). Probing the mechanism underlying this negative regulation, we find that HBV infection drives parallel upregulation of PD-L1 on liver-resident NK cells and PD-1 on intrahepatic T cells. PD-L1-expressing liver-resident NK cells suppress PD-1^hi^CD8^+^T-cells enriched within the HBV-specific response to therapeutic vaccination. Cytokine-activation of NK cells also induces PD-L1; combining cytokine activation with blockade of PD-L1 converts NK cells into efficient “helpers” that boost HBV-specific CD8^+^T-cell responses to therapeutic vaccination in mice and to chronic infection in humans. Our findings delineate a novel immunotherapeutic combination to boost the response to therapeutic vaccination in CHB and highlight the more widely applicable relevance of PD-L1-dependent regulation of T-cells by cytokine-activated NK cells.

## Introduction

Restoring an effective antigen-specific CD8^+^T-cell response is a primary goal of many immunotherapies being developed for chronic viral infections and malignancies. Therapeutic vaccines have shown limited success to date, but remain a key strategy as they promote priming and/or boosting of the endogenous immune response focused on the desired antigens. However, expansion of existing T cells is limited by their profound state of exhaustion, whilst newly-induced responses will be subjected to the same tolerogenic influences limiting their expansion and survival. Even the more immunogenic therapeutic vaccines currently in development will likely need to be accompanied by tailored immunotherapies to boost T cell responsiveness. For example, multiple HBV vaccines showing good immunogenicity in uninfected mice have shown less promising results once tested in models of CHB^1–3^. Similarly, the strong T cell responses induced by a ChAd3-HCV vaccine in uninfected humans were markedly attenuated when tested in donors with chronic HCV infection^4,5^.

The role of persistent antigen stimulation in driving epigenetic changes underpinning cell-intrinsic T cell exhaustion is well defined^6^. However, a better understanding of extrinsic negative regulation by neighbouring cell types relevant to the infection or tumour niche is needed. We and others have shown that human NK cells can regulate T cells directed against HBV in chronic infection, with the large population of liver-resident NK cells being particularly well-adapted to this role^7–10^. A negative regulatory role for NK cells through various pathways has similarly been demonstrated in mouse models of chronic viral infection^11–17^. Conversely, NK cells can have direct antiviral and anti-tumour activity as well as providing help to boost CD8^+^T-cells through their production of IFNγ^18–21^.

Here, we studied NK cell regulation of antigen-specific T cells *in vivo* to determine their dominant role in the response to therapeutic vaccination in a mouse model of CHB We found that liver-resident NK cells played a predominantly negative regulatory role, restraining vaccine-induced T cells against CHB through their upregulation of PD-L1. However, cytokine-activated NK cells could provide help to augment vaccine-induced HBV-specific CD8^+^T-cells, particularly once the restraining effect of their PD-L1 expression was blocked. The translational relevance of targeting PD-L1-dependent cytokine-activated NK cell regulation of HBV-specific CD8^+^T-cells was confirmed using human samples.

## Results

### Chronic HBV infection limits the antiviral response to ChAdOx1-HBV vaccination in mice

**Figure 1:**
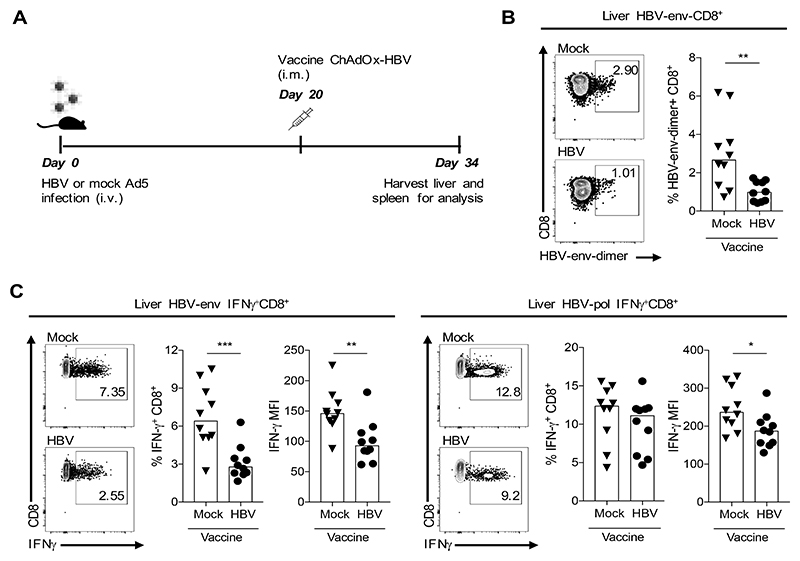
Intrahepatic CD8^+^T-cells show decreased functionality in chronic HBV infection. (A) C57BL/6 mice were infected with an adenoviral vector encoding the HBV genome (HBV) or a control empty vector (Mock). Mice were vaccinated with a chimpanzee adenoviral vector encoding HBV core, polymerase and surface antigens (ChAdOx1-HBV) 20 days after infection and organs were harvested 14 days after immunization (34 days after infection). (B) Proportion of HBV-envelope-specific CD8^**+**^T-cells identified by *ex vivo* staining using peptide-loaded MHC class I dimers, corresponding to the H-2K^b^-restricted immunodominant epitope from the envelope protein HBsAg (env190-197). (C) Frequency and MFI of IFNγ production by CD8^**+**^T-cells after overnight stimulation with overlapping peptides spanning HBV envelope or polymerase proteins followed by ICS. *, p<0.05 **, p<0.01; ***, p<0.001; p-values were determined by Mann-Whitney t-test. Bars represent median (n=5 mice per group, two experiments combined).

ChAdOx1-HBV is a multivalent vaccine consisting of a chimpanzee adenoviral vector encoding HBV core, polymerase and surface antigens that is highly immunogenic in uninfected mice^22^ and is currently being tested in Phase II trials in humans with CHB (EUDRACT number 2020-000190-25). We first used a mouse model to assess how CHB would alter the immunogenicity of the therapeutic vaccine *in vivo*. An adenoviral vector encoding the HBV genome (Ad-HBV) was injected intravenously to establish persistent low level HBV infection in the hepatocytes of immunocompetent C57BL/6 mice, as described previously^23,24^. Chronic infection was confirmed by the presence of circulating HBsAg 15 days after infection. Mock infection in control mice was achieved by intravenous injection of an empty adenoviral vector. Twenty days after establishment of CHB or mock infection, mice were immunized with the ChAdOx1-HBV vaccine intramuscularly and sacrificed 14 days later for analysis of HBV-specific T cell responses in liver and spleen (Fig.1a). Immunisation with chimpanzee adenovirus vectors did not increase IFNγ production when cells were stimulated with a peptide pool spanning the human adenovirus hexon protein, indicating there was no detectable induction of CD8^+^T-cell responses to the vector used in the infection (Suppl.Fig.1a).

Direct *ex vivo* analysis of CD8^+^T-cells specific for an H2K^b^-restricted epitope from HBsAg (env190-197) by dimer staining revealed significantly lower responses were induced by vaccination in HBV compared to mock-infected mice (mean 2.8 fold lower, Fig.1b). We then assessed CD8^+^T-cell functional responses in an unbiased manner following stimulation with overlapping peptides spanning the whole of the HBV surface and polymerase antigens (responses to core were undetectable, as previously reported with this vaccine formulation in this mouse strain^22^). Consistent with the results of the dimer staining, IFNγ, TNFα and CD107a production by HBsAg (env)-specific CD8^+^T-cells was significantly lower following vaccination of chronically infected compared to mock-infected mice (Fig.1c, Suppl.Fig.1b and c). By contrast, mice successfully resolving acute infection (following high dose Ad-HBV^24^) spontaneously mounted CD8^+^T-cell responses targeting HBsAg at higher frequencies than those with persistent HBV infection, that were not further boosted after vaccination (Suppl.Fig.1d). Polymerase-specific IFNγ-producing CD8^+^T-cells were induced by vaccination at higher frequencies than those against surface and were more subtly reduced by chronic compared to mock infection (trends to be reduced on all read-outs, only reaching significance for the quantity of IFNγ per cell and percentage of TNFα-producing cells, Fig.1c, Suppl.Fig.1b,c).

Thus, chronic HBV infection impaired the immune response to the ChAdOx1-HBV vaccine, with reduced frequencies and function of CD8^+^T-cells. The response to vaccination was more impaired in this model of CHB for T cells directed against HBsAg than the non-structural antigen polymerase, consistent with differing levels of tolerization of T cells against these antigens previously reported in HBV-transgenic mice and patients with CHB and therapeutic vaccination^25–29^.

### NK depletion enhances vaccine-induced HBV-specific T cells

**Figure 2:**
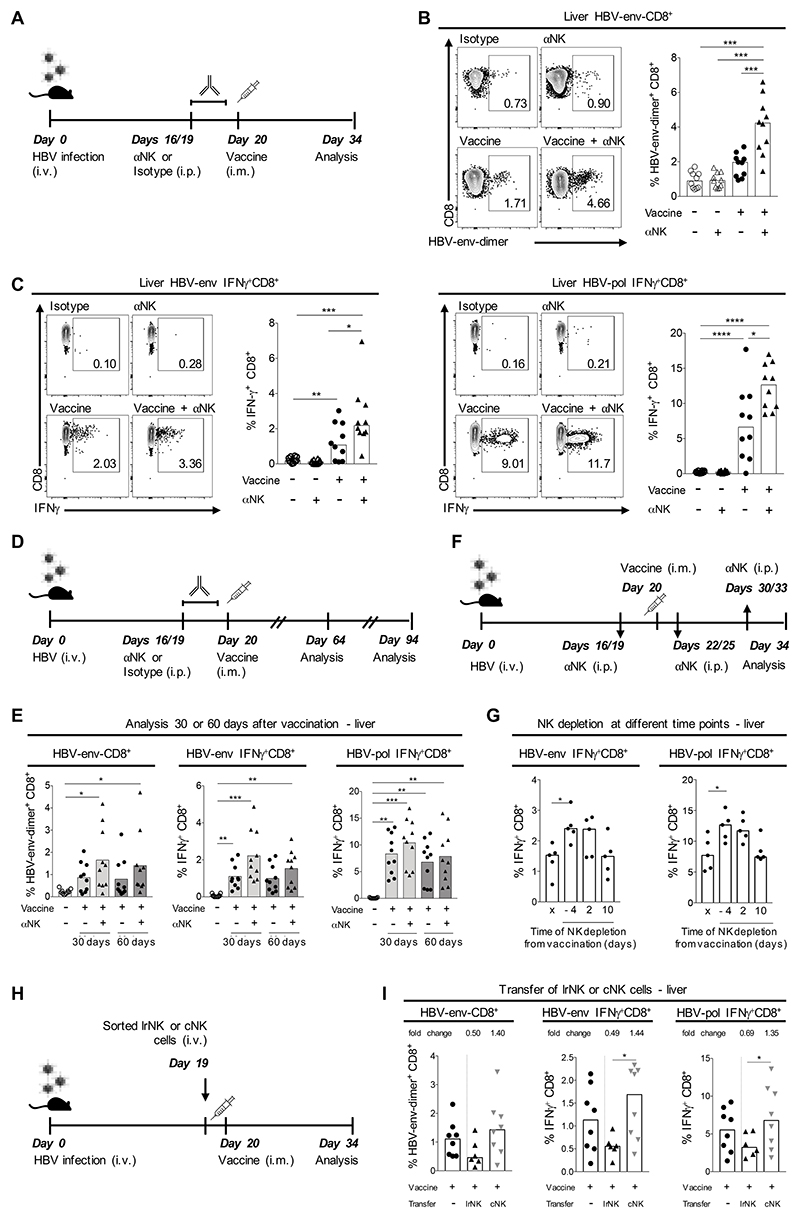
NK cells limit CD8^+^T-cell responses to therapeutic vaccination. (A) C57BL/6 mice were infected with an adenoviral vector encoding the HBV genome and treated with anti-NK1.1 (αNK) or isotype control antibody prior to therapeutic vaccination. Intrahepatic lymphocytes were harvested 14 days after immunisation (Day 34 after infection). At specific time points, HBV-envelope-specific CD8^**+**^T-cells were identified by *ex vivo* staining using H-2K^b^/env190-197 dimers or IFNγ production by CD8^**+**^T-cells was detected after overnight stimulation with overlapping peptides spanning HBV envelope or polymerase regions followed by ICS. (B) Representative plots and proportion of HBV-envelope-specific CD8^**+**^T-cells in liver 14 days after immunisation. (C) Representative plots and frequency of IFNγ-producing CD8^**+**^T-cells in liver 14 days after immunisation. (D) Mice were treated as described in A and immune responses were analysed 30 and 60 days after immunisation (Days 64 and 94 after infection). (E) Proportion of HBV-envelope-specific CD8^+^T-cells and detection of IFNγ production by CD8^+^T-cells 30 and 60 days after vaccination in liver. (F, G) Injection of anti-NK1.1 (αNK) or isotype control antibody started four days prior (-4), or 2 or 10 days after therapeutic vaccination and frequency of IFNγ-producing CD8^**+**^T-cells was assessed in liver 14 days after immunisation. (H, I) Liver-resident (lrNK) and conventional (cNK) NK cells were facs sorted from the liver of CD45.1 HBV-infected mice and transferred to congenic CD45.2 mice one day prior to therapeutic vaccination for assessment of HBV-envelope-specific CD8^+^T-cells and IFNγ production by CD8^+^T-cells (I; numbers on top show mean fold change comparing groups with transferred lrNK or cNK with mice without transfer). *, p<0.05; **, p<0.01; ***, p<0.001; ****, p<0.0001; p-values were determined by Kruskal-Wallis test (ANOVA) with a post hoc test for multiple comparisons (B-E) or Mann-Whitney t-test (H). Bars represent median. (B-E, n=5 mice per group, two experiments combined; F, one experiment performed; H, n=3-4 mice per group, two experiments combined.

Next, we tested the postulate that NK cells may be constraining the T cell response to the ChAdOx1-HBV vaccine in the setting of CHB. Groups of mice with persistent HBV infection (matched for levels of HBsAg) were assigned to have NK cells depleted or not, prior to ChAdOx1-HBV immunisation (Fig.2a). Anti-NK1.1 antibody was administered 4 days and 1 day prior to immunisation, using a low dose that preserved stable frequencies of invariant NKT cells (iNKT), as previously shown^12^, whilst successfully depleting over 90% of NK cells (Suppl.Fig.2a,b). This regimen was equally efficient at depleting conventional (DX5^+^; cNK) and liver-resident NK cells (CD49a^+^, lrNK also known as ILC1, Suppl.Fig.2b).

HBV infected mice that had NK cells depleted prior to immunisation showed a significantly increased population of H2Kb/env190-7 dimer CD8^+^T-cells in the liver compared to those with NK cell depletion or vaccine alone (Fig.2b). Within this expanded population of HBV-specific CD8^**+**^T-cells seen upon NK cell depletion, there was a skewing away from a central memory phenotype and towards effector memory and tissue-resident memory (T_RM_) subsets (Suppl.Fig.2c). NK cell depletion combined with vaccination not only drove the expansion of dimer-binding CD8^+^T-cells but also enhanced functional CD8^+^T-cells producing IFNγ after stimulation with envelope and polymerase peptide pools (Fig.2c). There was no significant increase in HBV-specific CD8 T cells following NK cell depletion in unvaccinated mice (Fig 2b, c). NK cell depletion did not increase the moderate liver inflammation induced by ChAdOx1-HBV immunisation (measured by ALT increase, Suppl.Fig.2d). NK cell frequencies were restored around 30 days after this antibody depletion strategy (Suppl.Fig.2e); as expected, the initial boost in vaccine-induced envelope-specific CD8^+^T-cell responses following NK cell depletion was therefore less pronounced by 30 and 60 days after immunisation, although still showed a non-significant trend to remain higher than in the non-depleted group (Fig.2d,e). More effective boosting of HBV-specific T cells was achieved by depletion of NK cells prior to, than after, immunisation, with a non-significant trend or no effect achieved by NK depletion starting 2 or 10 days after administration of the vaccine respectively (Fig.2f,g).

Augmentation of vaccine-induced HBV-specific CD8^+^T-cells was not observed following NK cell depletion in mock-infected (Ad-empty) mice (Supp.Fig.2f). Likewise, NK cell depletion did not increase vaccine-induced antigen-specific CD8^+^T-cell responses in the spleen (Suppl.Fig.2g), suggesting that NK cell-mediated regulation of CD8^+^T-cell responses to this hepatotropic infection was localised to the liver. We therefore purified liver-resident and conventional NK cell subsets from CHB mice and adoptively transferred these to CHB mice one day before immunisation (Fig.2h). Using the congenic marker CD45.1, we confirmed that adoptively transferred liver-resident NK cells selectively homed to the liver whereas CD45.1 conventional NK cells localized in both liver and spleen (Supp.Fig.2h). Transfer of small numbers of liver-resident NK cells was able to suppress the HBV-specific T cell response to immunisation, which conversely tended to be boosted by transfer of equivalent numbers of conventional NK cells (Fig.2i). Taken together these data pointed to a role for a pathway induced locally in the chronically HBV-infected liver that was able to render vaccine-induced CD8^+^T-cells susceptible to regulation by liver-resident NK cells.

### Parallel upregulation of the PD-1 pathway on liver-resident NK cells & T cells in HBV infection

**Figure 3:**
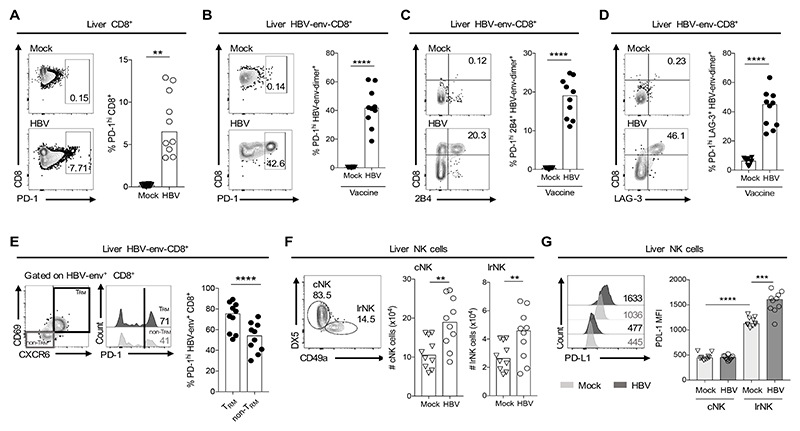
HBV infection induces modulation of the PD-1/PD-L1 axis. C57BL/6 mice were infected with an adenoviral vector encoding the HBV genome (HBV) or a control empty vector (Mock) and vaccinated 20 days after infection. Organs were harvested for analysis 14 days after immunization (34 days after infection). (A) Representative example and frequencies of PD-1^hi^ global CD8^+^T-cells in liver from unvaccinated mice. (B) Example plot and proportion of envelope-specific (gated on H-2K^b^/env190-197 dimer-stained CD8^+^T-cells) in vaccinated mice. (C,D) Percentages of env-specific PD-1^hi^ intrahepatic CD8^+^T-cells expressing 2B4 (C) or LAG-3 (D). (E) Representative example and frequencies of PD^hi^ HBV-envelope-specific CD8^+^T-cells on liver-resident (CD44^hi^CD69^+^CXCR6^+^, T_RM_) or non-resident liver-infiltrating memory (CD44^hi^CD69^-^CXCR6^-^, non-T^RM^) CD8^+^T-cells. (F) Representative example and numbers of DX5^+^ conventional (cNK) or CD49a^+^ liver-resident (lrNK) NK cells. (G) Representative example and expression of PD-L1 (MFI) on intrahepatic conventional (cNK) or liver-resident (lrNK) NK cells. **, p<0.01; ***, p<0.001; ****, p<0.0001; p-values were determined by Mann-Whitney t-test (A-F) or Kruskal-Wallis test (ANOVA) with a post hoc test for multiple comparisons (G). Bars represent median (n=5 mice per group, two experiments combined).

Next, we examined CD8^+^T-cells and NK cells for relevant inhibitory pathways that were activated on both cell types upon HBV infection in a liver-compartmentalised manner. We have previously shown that the co-inhibitory receptor PD-1 is highly expressed on human liver-resident CD8^+^T-cells and further upregulated in the context of CHB^30^ which can constrain intrahepatic HBV-specific CD8^+^T-cells in subjects with CHB^31^. We observed a selective expansion of global CD8^+^T-cells within the liver (and not the spleen) that likewise included a fraction that expressed high levels of PD-1, induced by day 7 and maintained for at least 60 days following establishment of persistent HBV infection in mice (Fig.3a, Supp.Fig.3a-c). These global PD-1^hi^CD8^+^T-cells did not have higher activation levels (CD44) than those in mock-infected mice but co-expressed the co-inhibitory receptors 2B4 and LAG-3 (Supp.Fig.3d-f), consistent with an exhausted phenotype characteristic of chronic viral infections^32^.

Importantly, HBV envelope-specific CD8^+^T-cells induced by ChAdOx1-HBV immunisation (identified *ex vivo* by H2K^b^/env190-197 dimer staining) showed a particularly marked upregulation of PD-1 (mean 37.4% PD-1^hi^ compared to 8.9% on global dimer-negative CD8^+^T-cells); this was only seen upon immunisation on the background of chronic HBV infection and not in mock-infected mice (Fig.3b). A proportion of PD-1^hi^ HBV-specific CD8^+^T-cells induced by vaccination of CHB mice co-expressed the inhibitory receptors 2B4 and particularly LAG-3, that were again not induced in mock-infected mice (Fig.3c,d). Consistent with PD-1^hi^ CD8^+^T-cells being a feature of chronic infection, we found that global and HBV-specific CD8^+^T-cells expressed only low or intermediate levels of PD-1 following self-resolving infection (Suppl. Fig. 3g). PD-1 was induced on IFNγ–producing CD8^+^T-cells targeting both envelope and polymerase, but at higher levels on env-specific, consistent with more profound exhaustion of this specificity (Supp.Fig.3h). An analogous upregulation of PD-1 was not seen on splenic CD8^+^T-cells in HBV-infected mice (Supp.Fig.3i). In line with this being a liver-localised effect, PD-1 upregulation was more marked on liver-resident (CD44^hi^CD69^+^CXCR6^+^) than non-resident liver-infiltrating (CD44^hi^CD69^-^ CXCR6^-^) HBVenv-specific CD8^+^T-cells in HBV-infected mice (Fig.3e).

To investigate whether the induction of PD-1 on CD8^+^T-cells could be a relevant receptor for NK cell regulation, we examined expression of the ligand PD-L1 on conventional and liver-resident NK cells upon HBV infection. Mice persistently infected with HBV had an expansion in absolute numbers of both conventional and liver-resident NK cells in the liver compared to mock-infected mice, whereas splenic NK cells numbers remained stable (Fig.3f, Supp.Fig.3j). PD-L1 was preferentially expressed by liver-resident NK cells and was selectively further upregulated on this subset (but not on conventional liver or splenic NK cells) in HBV-infected mice, remaining at higher levels for at least 30 days after infection (Fig.3g, Supp.Fig.3k,l. We examined several other potentially relevant NK ligands (CD48 and MHC-II, respectively binding the co-inhibitory receptors 2B4 and LAG3 upregulated on PD-1^hi^CD8+T-cells in this model; NKG2D and TRAIL because of their induction on liver-resident NK cells in human CHB^7,8,10^); none of these were selectively further upregulated on murine liver-resident NK cells by HBV infection (Supp.Fig.3m-p).

In summary, we observed that persistent infection in the Ad-HBV model drove PD-1 expression on CD8^+^T-cells localised within the liver, including the virus-specific fraction induced by therapeutic vaccination, in tandem with selective upregulation of PD-L1 confined to the liver-resident subset of NK cells.

### PD-L1-dependent NK cell regulation of vaccine-induced T cells

**Figure 4:**
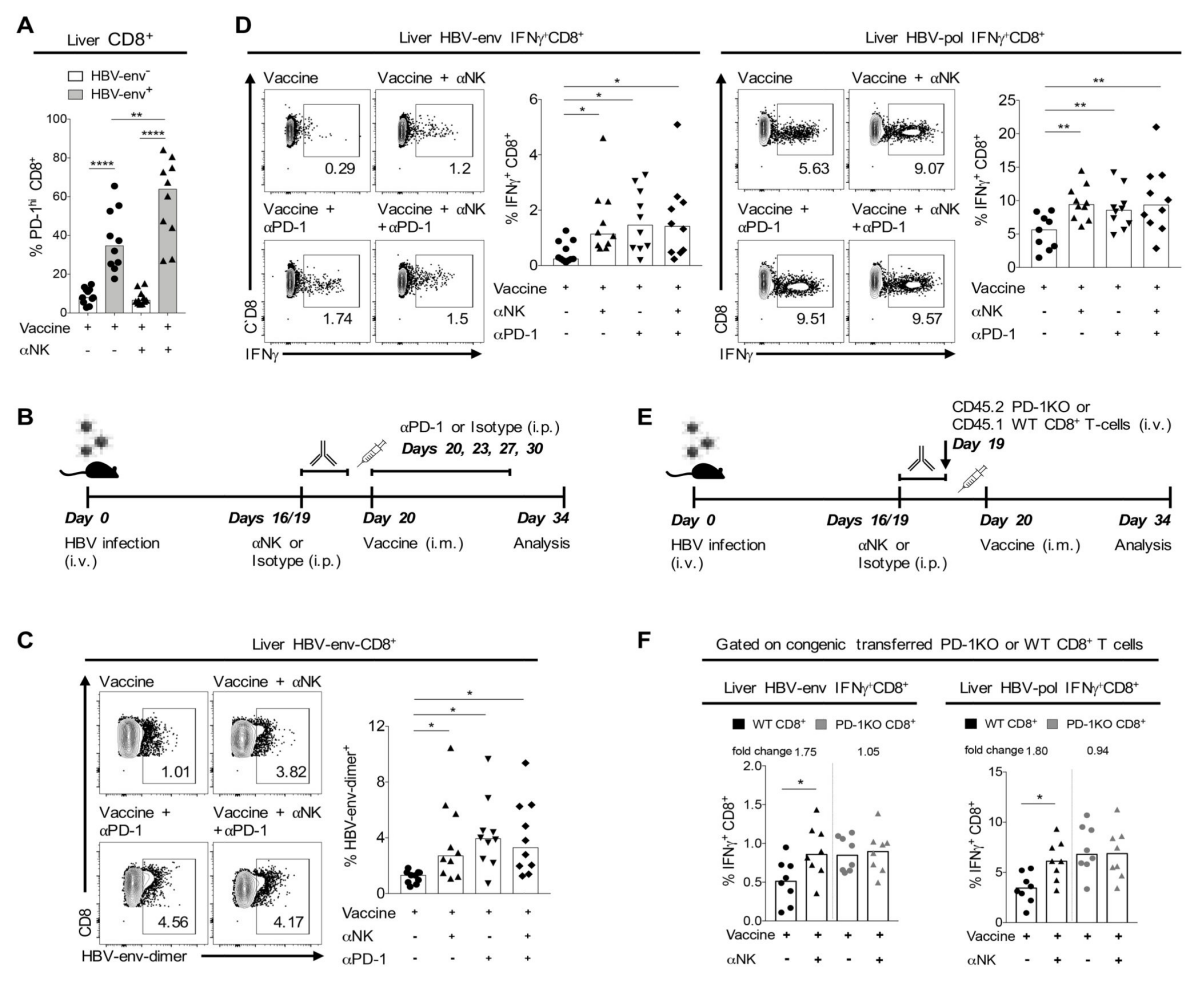
PD-L1 plays a role in NK cell regulation of CD8^+^T-cells. (A) Frequencies of env-specific (HBV-env^+^) and env-negative (HBV-env^-^) PD-1^hi^ intrahepatic CD8^+^T-cells. (B) C57BL/6 mice were infected with an adenoviral vector encoding the HBV genome and vaccinated 20 days after infection. Groups of mice were treated with anti-NK1.1 and/or anti-PD-1. (C) Representative plots and proportion of HBV-envelope-specific CD8^+^T-cells identified by *ex vivo* staining using H-2K^b^/env190-197 dimers. (D) Detection of IFNγ production by CD8^+^T-cells after overnight stimulation with overlapping peptides spanning envelope or polymerase regions of HBV followed by ICS. (E) CD8^+^T-cells isolated from the spleen of CD45.2 PD-1KO or CD45.1 wild-type (WT) mice were transferred to opposite congenic mouse recipient one day prior to therapeutic vaccination +/-NK depletion. (F) Proportion of transferred PD-1 or WT CD8^+^T-cells in liver producing IFNγ after HBV-envelope or polymerase peptide stimulation (mean fold change comparing IFNγ production by transferred CD8^+^T-cells in NK depleted *vs*. non-depleted mice). *, p<0.05; **, p<0.01; ****, p<0.0001; p-values were determined by Kruskal-Wallis test (ANOVA) with a post hoc test for multiple comparisons (A, C, D) or Mann-Whitney t-test (F). Bars represent median (n=4-5 mice per group, two experiments combined).

Based on the induction of the PD-1 axis on liver-resident NK cells and CD8^+^T-cells in HBV, we next sought evidence for a functional role of this pathway *in vivo*. We reasoned that if PD-L1-expressing liver-resident NK cells were selectively targeting T cells expressing the highest levels of PD-1, then NK cell depletion would preferentially rescue PD-1^hi^CD8^+^T-cells. In line with this, we noted that HBV-specific CD8^+^T-cells induced by immunisation following NK cell depletion were enriched for high PD-1 expression compared to those generated in the presence of NK cells (Fig.4a). To further investigate the contribution of the PD-L1/PD-1 axis to NK cell regulation in this setting, we treated vaccinated mice with four doses of anti-PD-1 antibody starting at the same day as immunisation, combined or not with NK depletion (Fig.4b). PD-1 blockade recapitulated the rescue of vaccine-induced HBV-specific CD8^+^T-cells (dimer-staining and IFNγ production) seen with NK cell depletion, however, the addition of NK cell depletion to anti-PD-1 blockade did not further augment this increase (Fig.4c,d). Similarly, NK cell depletion or PD-1 blockade combined with therapeutic vaccination induced comparable partial control of viral load that was not further improved when these treatments were combined (Suppl.Fig.4a). This redundancy between PD-1 blockade and NK cell depletion supported a central role for the PD-1 axis in NK cell regulation of vaccine responses in the HBV-infected liver. To confirm a functional role for the PD-1 pathway in NK cell regulation of anti-HBV T cells, congenic CD8^+^T-cells from PD-1 KO or wild-type mice were transferred to CHB mice with or without NK depletion (Fig.4e, Suppl. Fig.4b). Transferred WT CD8^+^-T cells rapidly upregulated PD-1 in the chronically HBV-infected liver and showed reduced HBV-specific IFNγ production in the presence of NK cells. On the other hand, transferred PD-1KO CD8 T cells did not increase IFNγ production after NK depletion but had baseline HBV-specific responses equivalent to those in the NK cell depleted mice (Fig.4f). Thus, HBV-specific T cells induced by therapeutic vaccination in CHB mice required PD-1 expression in order to be restrained by NK cells.

### PD-L1 blockade enhances helper function of cytokine-activated NK cells in murine and human CHB

**Figure 5:**
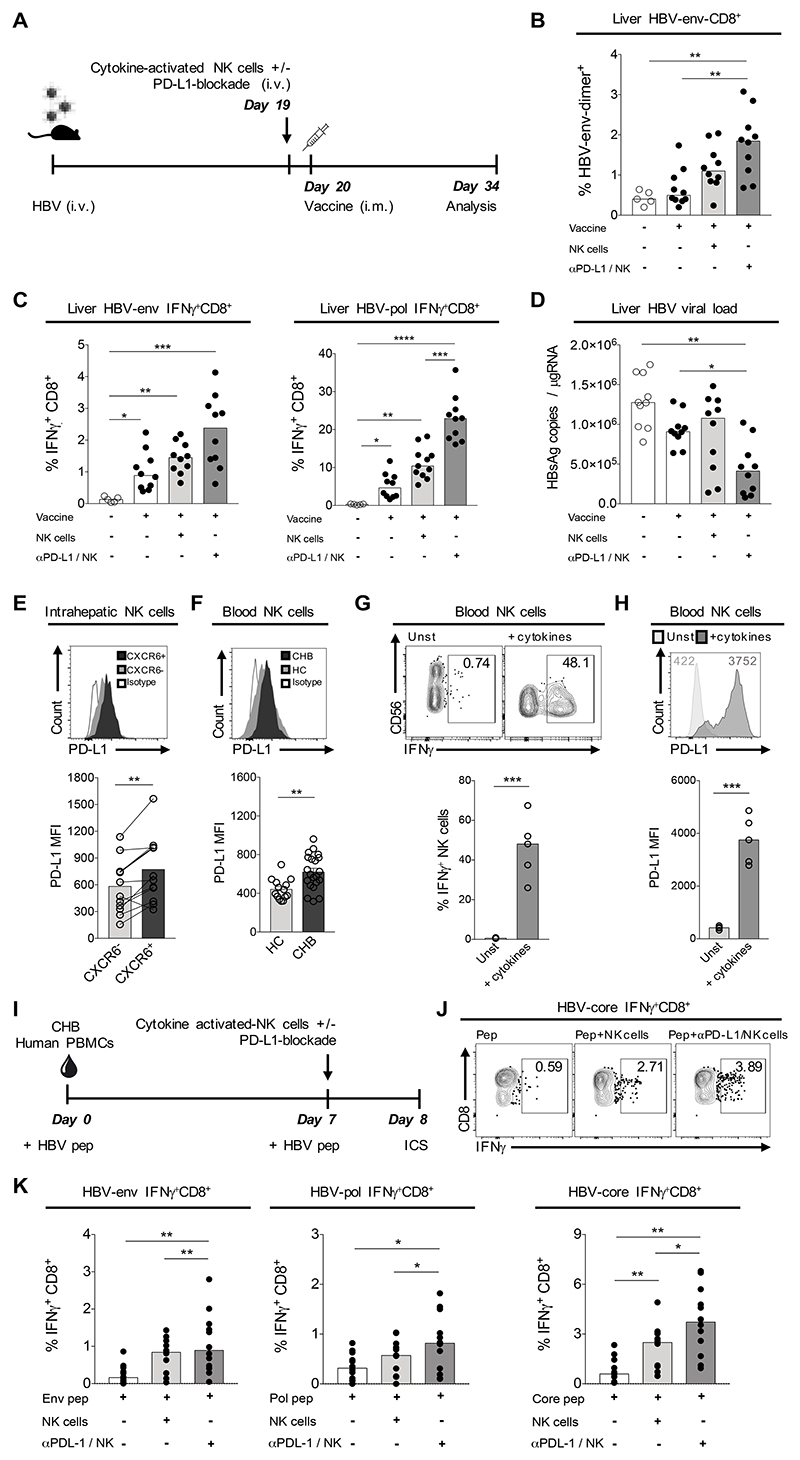
PD-L1 blockade enhances helper function of cytokine-activated NK cells. (A) Murine splenic cytokine-treated NK cells were incubated with PD-L1-blocking or isotype control antibody and transferred to persistently infected mice one day prior to therapeutic vaccination. (B) Frequencies of HBV-envelope-specific CD8^+^T-cells identified by *ex vivo* H-2K^b^/env190-197 dimer staining. (C) Detection of IFNγ production by CD8^+^T-cells after overnight stimulation with overlapping peptides spanning HBV envelope or polymerase regions followed by ICS. (D) Quantitative real-time PCR analysis of HBsAg mRNA extracted from the liver of infected mice (B-D, n=5 mice per group, two experiments combined). (E) Human liver NK cell expression of PD-L1 on liver-infiltrating (CXCR6^-^) and liver-resident (CXCR6^+^) NK cell subsets from surgical resections (non-diseased associated tissue margins of colorectal liver metastases, n=6) and perfusion liquid (n=6). (F) Expression of PD-L1 on NK cells in peripheral blood from healthy donors (n=14) (HC) or patients with chronic HBV infection (n=21) (CHB). (G, H) Representative plot of IFNγ production (G) or PD-L1 expression (H) after stimulation of human NK cells from peripheral blood with IL-2, IL-12, IL-15 and IL-18 (+cytokines) compared to unstimulated (Unst) NK cells (n=5). (I) PBMCs from patients with CHB were expanded for 7 days in the presence of HBV envelope, polymerase or core overlapping peptides and re-stimulated overnight in the presence of autologous activated NK cells that were previously treated with PD-L1-blocking antibody or isotype control. Detection of IFNγ production by CD8^+^T-cells after stimulation with overlapping peptides spanning HBV core (J), envelope or polymerase regions (K) (n=8). Bars represent median. *, p<0.05; **, p<0.01; ***, p<0.001; ****, p<0.0001; p-values were determined by Kruskal-Wallis test (ANOVA) with a post hoc test for multiple comparisons (B-D, J,K), Wilcoxon’s paired t-test (E) or Mann-Whitney t-test (F-H).

Immunomodulatory cytokines able to boost the antiviral potential of T and NK cells are being evaluated in combination with therapeutic vaccines for the functional cure of CHB^1,3^. Infusions of NK cells pre-activated with cytokines are another promising therapeutic strategy in cancer and infection. Although cytokines may boost direct antiviral effects of NK cells and their capacity to provide T cell help through IFNγ, we postulated cytokines may also drive negative NK cell regulation of antiviral T cells^1,19– 21^. We therefore examined whether the potential for cytokine-activated NK cells to support the T cell response to therapeutic vaccination would be limited by PD-L1 induction. *In vitro* stimulation of splenic NK cells with activatory cytokines (IL-2, IL-12, IL-15, IL-18) resulted in a simultaneous increase in their production of IFNγ (Supp.Fig.5a, expected to provide help to CD8^+^T-cells) and expression of PD-L1 (Supp.Fig.5b, postulated to restrain vaccine-induced CD8^+^T-cells). Cytokine-activated NK cells were blocked with anti-PD-L1 or an isotype control antibody *in vitro* and transferred to HBV-infected mice before ChAdOx1-HBV immunisation (Fig.5a). Mice that received cytokine-activated NK cells had comparable, or a slight tendency towards increased, frequency and function of HBV-specific CD8^+^T-cells induced by immunisation compared to those with vaccine alone. However, when cytokine-activated NK cells were pre-treated with PD-L1-blocking antibodies, they supported significantly greater HBV-specific CD8^+^T-cell responses to the vaccine that was lost after treatment of mice with IFNγ-blocking antibody (Fig.5b,c and Suppl.Fig.5c,d). The transfer of PD-L1 blocked, cytokine-treated NK cells promoted an immune response better able to control viraemia (Fig.5d). These data revealed that cytokine-treated NK cells can boost vaccine-induced HBV-specific CD8^+^T-cells in an IFNγ-dependent manner, particularly if their negative regulatory effect is removed by PD-L1 blockade.

To investigate the translational relevance of this mechanism we tested whether human NK cells also had the capacity to express PD-L1 and regulate HBV-specific T cells through this axis. We, and other groups, recently identified CXCR6 as a useful marker for human liver-resident NK cells^10,33,34^. We therefore isolated intrahepatic lymphocytes from non-diseased associated tissue of liver resections or from pre-transplant perfusion liquid and stained the liver-resident (CXCR6^+^) and liver-infiltrating (CXCR6^-^) NK cells for PD-L1. We found that healthy human intrahepatic NK cells constitutively expressed PD-L1 and, as in mice, this was more marked on the liver-resident subset (Fig.5e). PD-L1 was barely detectable on NK cells from the blood, but was significantly increased on those circulating in patients with CHB, particularly on the CD56^bright^NK cell subset (which has overlapping features with liver-resident NK cells, Fig.5f and Suppl.Fig.5e). Similar to murine splenic NK cells, human peripheral blood NK cells showed a potent upregulation of IFNγ production upon cytokine activation (Fig.5g). Cytokine activation increased NK cell granzyme and perforin but did not significantly induce cytotoxic degranulation, whereas PD-L1 expression was markedly upregulated (Suppl.Fig.5f and Fig.5h).

We then expanded HBV-specific CD8^+^T-cells by stimulating PBMC from donors with CHB using peptides spanning the HBV core, envelope and polymerase proteins, with or without the addition of autologous cytokine-activated NK cells (Fig.5i). The addition of cytokine-activated NK cells increased HBV-specific CD8^+^T-cells (Fig.5j,k) and this augmentation was partially abrogated by IFN-γ-blocking antibodies (Suppl.Fig.5g). Similarly, the boosting effect of supernatants harvested from cytokine-activated NK cells was reduced by IFN-γ-blockade (Supp.Fig.5h). Although we have demonstrated roles for TRAIL and NKG2D in constitutive NK cell regulation of T cells, blockade of these pathways did not enhance the helper effect of cytokine-activated NK cells (Suppl.Fig.5i). However, consistent with the marked upregulation of PD-L1 on cytokine-activated NK cells, blockade of this ligand unleashed more effective NK cell help, resulting in a significantly larger expansion of HBV-specific CD8^+^T-cells directed against each of the viral proteins (Fig.5j,k). PD-L1 blockade was unable to augment NK cell help to T cells once these populations were separated by a transwell, consistent with contact-dependent negative regulation by NK cell PD-L1 (Suppl.Fig.5j). Thus, human cytokine-treated NK cells could drive the expansion of functional HBV-specific CD8^+^T-cells through provision of IFNγ, particularly when their PD-L1-dependent restraining effects were blocked.

## Discussion

New immunotherapeutic regimens are urgently needed to enhance the T cell response to therapeutic vaccines in chronic viral infections and cancer. We have identified a novel role for NK cells in restraining the antiviral CD8^+^T-cell response to therapeutic vaccination. Depleting NK cells *in vivo* immediately prior to therapeutic vaccination enhanced the expansion of functional virus-specific CD8^+^T-cells, with beneficial effects sustained for at least two months. We found that selective expression of PD-L1 by liver-resident NK cells was upregulated upon chronic HBV infection. In tandem, PD-1 was induced on intrahepatic CD8^+^T-cells, with a striking enrichment of PD-1^hi^CD8^+^T-cells in the HBV-specific responses induced by therapeutic vaccination. NK regulation preferentially targeted PD-1^hi^ HBV-specific CD8^+^T-cells, was redundant with PD-1 blockade, and was abrogated by PD-1 knockdown *in vivo*, in keeping with a dominant role for this pathway in NK cell suppression of vaccine-induced T cells in this CHB model. NK cells that were adoptively transferred after they had been treated with cytokines and PD-L1 blocking antibodies became predominant “helpers”, boosting functional HBV-specific CD8^+^T-cells induced by therapeutic vaccination. Crucially, we discovered human NK cells were also capable of expressing PD-L1, particularly the liver-resident fraction, and could upregulate it upon HBV infection or cytokine activation. Blockade of PD-L1 on cytokine-activated human NK cells promoted their capacity to enhance the *in vitro* expansion of HBV-specific CD8^+^T-cells from donors with CHB.

NK cells are increasingly recognised to have potent positive or negative regulatory capacity in addition to their direct antiviral and anti-tumour functionality. A better understanding of the settings where NK cells calibrate T cells, and the pathways utilised, will allow selective immunotherapeutic harnessing of their beneficial roles and blockade of their suppressive effects. NK cells are particularly abundant within the liver and include a specialised, transcriptionally distinct tissue-resident fraction, considered analogous to ILC1s^35^. Intrahepatic NK cells are well-positioned within the narrow-lumen, low-flow sinusoidal vasculature to come into prolonged contact with resident and recirculating T cells^7,10,30^. Congruent with this, we found that HBV vaccine-induced T cells were inhibited locally in the liver but not in the spleen, an effect recapitulated by adoptive transfer of liver-resident but not conventional NK cells. Constitutive expression and infection-induced upregulation of PD-L1 by liver-resident NK cells implicated this pathway in the tight regulation of T cell responses characteristic of this tolerogenic organ. Thus, regulation of highly activated and/or exhausted T cells may constitute a homeostatic role for liver-resident NK cells; whilst this would be beneficial in preventing pathological overzealous reactions to acute insults, it represents a counterproductive restraint on the optimal expansion of vaccine-boosted T cell responses to a chronic hepatotropic infection. Induction of PD-L1 on liver-resident NK cells has similarly been recently described in acute adenovirus and chronic LCMV infection^36^ but the mechanism underpinning its upregulation remains undefined. The liver environment also contributes to PD-1 expression on T cells (observed on congenically transferred T cells and endogenous non-antigen-specific intrahepatic T cells following infection, as seen in human CHB) which is then more markedly upregulated on those T cells stimulated by cognate antigen in chronic but not acute CHB. The mouse model we used has a number of limitations^37^, including lack of cccDNA formation and viral spread, but to some extent mimics the state of low-level HBV replication without liver inflammation seen in many human low-level carriers, manifesting an exhausted PD-1^hi^ T cell response to therapeutic vaccination particularly affecting the envelope response. It would be interesting to test whether a model with higher levels of HBV replication and/or inflammation would drive further accumulation of PD-L1-expressing NK cells.

Although the restraining effects of NK cells have been demonstrated in a number of other settings, they have not previously been shown to limit the CD8^+^T-cell response to therapeutic vaccination. NK cells have, however, been shown to inhibit CD4 Tfh responses, resulting in impaired affinity maturation of antibody responses in a murine model of prophylactic immunisation, effects that would also be relevant to study in HBV therapeutic vaccination. The CD8^+^T-cells we found to be preferentially rescued by NK cell depletion following therapeutic vaccination expressed high levels of PD-1 and other co-inhibitory molecules, yet resulted in enhanced antiviral functionality. This apparent discrepancy is in line with accumulating data showing that T cells with an “exhausted” profile can still make a critical contribution to viral control. We also found a preferential increase in CD8^+^T_RM_ following NK cell depletion, consistent with their high expression of PD-1, thought to restrain them in homeostatic conditions whilst allowing rapid functionality upon TCR engagement and in line with their association with HBV control^30^.

The translational relevance of our study was underscored by the finding that PD-L1 was also preferentially expressed by human liver-resident NK cells. Moreover PD-L1 could be detected on NK cells sampled directly *ex vivo* from the circulation of patients with CHB or, more strikingly, after cytokine activation. Using samples from CHB patients, we observed that PD-L1 blockade of cytokine-stimulated NK cells enhanced HBV-specific CD8^+^T-cell expansion, reinforcing the potential of this as a therapeutic strategy for the functional cure of CHB. Whereas we and others previously identified roles for TRAIL and NKG2D in constitutive NK cell regulation of human HBV-specific T cells^7–9^, these pathways did not dominate in the setting of cytokine-activated NK cells. Instead, we found that PD-L1 is a major mechanism of restraint by cytokine-activated human NK cells, that can expand HBV-specific CD8^+^T-cells in an IFNγ-dependent manner. PD-1 blockade is already being tested as a way of enhancing the T cell response to therapeutic vaccination in patients with HBV, following promising results in the woodchuck hepatitis virus model^3,38,39^; uncovering the contribution of liver-resident NK cells to the PD-L1 axis provides new ways of interrupting negative regulation in this setting. Further studies are needed to see whether PD-1^hi^CD8^+^T-cells may be marked out for NK cell targeting by the upregulation of additional receptors that could be targeted therapeutically in addition to the PD-L1/PD1 axis. Our data suggest the optimal timing for blocking such negative regulation is around the time of therapeutic vaccination rather than in the weeks following it, but that the benefits can be durable. In addition to *in vivo* blockade of NK cell negative regulation, our data raise the possibility of administering cytokine-activated, PD-L1 blocked NK cells or CAR-NK cells as a liver-homing adjuvant to therapeutic vaccination in CHB.

Since PD-L1 has also been shown to be induced on NK cells infiltrating tumours and tumour-draining lymph nodes in a mouse model^40^, further exploration of its role in constraining anti-tumour T cells in human liver cancers is now warranted; our recent demonstration of high frequencies of liver-resident NK cells in human hepatocellular carcinoma (HCC)^41^ raises the possibility they can utilise PD-L1 to inhibit local T cells in this setting. In addition, cytokine activation of adoptively transferred NK cells and chimeric antigen receptor (CAR)-NK cells is being widely tested as a way of boosting NK cell anti-tumour functionality^42^; for example, we recently showed that IL-15 can boost NK cell functionality against HCC *in vitro*^41^. Our new finding that these activatory cytokines also drive PD-L1 expression on NK cells provides a rationale for the addition of PD-L1 blockade or knockdown, in order to preserve the beneficial potential of NK cells, whilst avoiding their detrimental inhibition of PD-1^hi^CD8^+^T-cells. Thus, our data demonstrate an immunotherapeutic combination able to enhance the efficacy of therapeutic vaccination in CHB, whilst exemplifying more generically how defining mechanisms of calibration of antigen-specific CD8^+^T-cells by NK cells allows them to be harnessed to act synergistically rather than in opposition.

## Materials and Methods

### Study design

The primary objective of our study was to elucidate NK cell regulation of antigen-specific CD8^+^T-cell responses in a murine model of persistent HBV infection. Mice infected by injection of an adenoviral vector carrying the HBV genome were matched on the basis of their serum levels of the HBV envelope antigen (HBsAg) persisting at day 15 before being assigned to receive therapeutic HBV immunisation with or without NK cell depletion. Having observed changes in the PD-1 axis upon HBV infection and NK cell depletion, we then developed the study to further investigate the role of this pathway in mediating NK cell regulation of HBV-specific T cells, treating mice with PD-1 blocking mAbs, transferring PD-1KO CD8^+^T-cells or NK cells pre-treated with anti-PD-L1. Mouse spleens, liver and blood were collected and cells were harvested and stained for flow cytometry. Animal experiments were performed in groups of 5 based on previous experiments and publications using the same or similar models. Investigators were not blinded for measurements. All the experiments were performed at least twice. All animal studies were performed under approved protocols of the Institutional Animal Care and Use Committee and the institutional review board. All the mouse tissues were obtained following institutional ethical guidelines of University College London and authorized by the United Kingdom Home Office.

The relevance of PD-L1-mediated regulation of CD8^+^T-cells was then confirmed in human samples based on existing ethical approval to study HBV immunology (including NK cell/T cell interactions). Written informed consent was obtained from all donors and the study was approved by the relevant ethics boards detailed below. We isolated NK cells from unaffected liver margins of patients undergoing tumor resections and from pre-transplant perfusion liquid to analyse *ex vivo* PD-L1 expression on intrahepatic liver-resident versus liver infiltrating NK cells. We isolated PBMCs from healthy donors or CHB patients to compare PDL1 on their NK cells and to then investigate whether blocking PD-L1 on cytokine-activated NK cells would increase their capacity to boost HBV-specific CD8^+^T-cell responses using peptide-expanded PBMCs from CHB patients. The number of samples for each experiment is listed in accompanying figure legends. No outliers were excluded from any experiments or analyses reported in this manuscript.

### Mice

Male 6-8 weeks old C57BL/6, CD45.1 (inbred C57BL/6) and CD45.2 PD-1KO mice were purchased from Envigo (UK) or Charles Rivers (Italy) and maintained in individually ventilated cages in a containment level 2 animal facility at the University College London. All experiments were approved by the Animal Welfare and Ethical Review Body of University College London and authorized by the United Kingdom Home Office (PPL 70/8145).

### Vectors

Induction of persistent infection was achieved after transfer of HBV genotype D genome to the liver of mice using an Adenovirus 5 vector. For this, the HBV1.3 genome was inserted into the E1 region of adenovirus (Ad5E1/E3) backbone plasmid pAd/PL-DEST through recombination following the manufacturer’s instructions (Gateway System; Invitrogen, Karlsruhe, Germany)^24^. The empty adenovirus vector was used as control. The adenoviral vectors genomes were transfected into human embryonic kidney 293 cells for production of vectors and purified by cesium chloride (CsCl) gradient centrifugation. For the construction of the therapeutic vaccine, HBV genotype C pre-core (PreC), core, non-functional polymerase (Pmut), large surface antigen and class II shark invariant chain (SIi) sequences were cloned into the chimpanzee adenovirus vector ChAdOx1 under the control of CMV promoter^22^. Vaccines were produced using the T-Rex-293 cell line (Thermo Fisher Scientific, Paisely, UK) and purified by CsCl centrifugation at the Viral Vector Core Facility (VVCF) of the Jenner Institute, University of Oxford, UK.

### Mouse experiments and HBV Infections

For Ad-HBV chronic infection, 3×10^8^ infectious units (iu) in 100 μL of saline were delivered into mice intravenously. In selected experiments, self-resolving infection was induced after injection of 3×10^9^ infectious units (iu)^24^ of Ad-HBV. Blood specimens for detection of HBsAg and ALT were collected 15 days after infection or at experiment endpoint. Mice were vaccinated with ChAdOx1-HBV (ChAd-HBV) 20 days after infection via intramuscular injection into the hind leg with chimpanzee-adenovirus (5×10^7^ infectious units (iu) in 50 μL) and euthanised 14, 30 or 60 days after immunisation. In some experiments, mice received anti-NK1.1 monoclonal antibody (PK136) or control mouse IgG2a (C1.18.4) (Bio-X-Cell) at days 16 and 19 after infection (25μg/dose i.p.) (Day -4 from immunisation). Alternatively, anti-NK1.1 injection started 2 or 10 days after vaccination. In selected experiments, mice were treated with 4 doses of 100 μg of anti-PD-1 (29F.1A12, BioXcell) or IgG2a isotype control given 3-4 days apart starting 20 days after infection.

### Cell transfer experiments

CD8^+^T-cells were transferred after being isolated from spleens of CD45.1 WT or CD45.2 PD-1KO mice by negative magnetic sorting (Miltenyi Biotec) and 500,000 cells were transferred intravenously to HBV infected mice one day prior to vaccination. Intrahepatic NK cells from HBV infected CD45.1 mice were isolated by negative magnetic sorting (Miltenyi Biotec) followed by flow cytometry cell sorting of conventional NK (DX5^+^) or liver-resident NK cells (CD49a^+^) and 200,000 cells were transferred intravenously one day prior to vaccination. Alternatively, NK cells from spleens of CD45.1 mice were purified by negative magnetic sorting (Miltenyi Biotec). NK cells were stimulated for 16h with mouse IL-2 (200U/mL), IL-12 (50ng/mL), IL-15 (50ng/mL) and IL-18 (50ng/mL) (Peprotech). Cells were treated with anti-PD-L1 (10μg/mL) (10F.9G2, Biolegend) or isotype control for 1h. Cells were washed 3 times and 200,000 cells were transferred intravenously. In selected experiments, mice were treated with 2 doses of 300μg of IFNγ blocking antibody (XMG1.2, Biolegend) intraperitoneally on the same day and 7 days after immunisation.

### Mouse splenocyte and intrahepatic lymphocyte isolation

Spleen and livers were collected from mice in phosphate buffered saline (PBS) and lymphocytes were isolated by gentle mechanical disruption through a 70 μm cell strainer (Greiner) followed by one-minute exposure to ACK lysis buffer (Life Technologies). Prior to red blood cell lysis, intrahepatic lymphocytes were isolated using 30% Percoll (GE healthcare) density gradient centrifugation.

### Detection of HBV-Specific CD8^+^T-Cells by Dimer Staining

Murine envelope-specific CD8^+^T-cells were detected using soluble DimerX H-2K^b^:Ig fusion protein technology (BD Biosciences) according to the manufacturer’s instructions. Briefly, 0.8 µg dimer per sample was loaded with 2.4 μg H-2K^b^-restricted HBsAg-derived epitope peptide env190-197 (VWLSVIWM) at 4°C for 24 h. Cells were firstly incubated with purified anti-mouse CD16/32 antibody (Biolegend) to block their FcRs at 4°C for 10 min, and then were incubated with peptide-loaded or unloaded dimer at 4°C for 1 h. PE-anti-mouse IgG1 antibody was used to label the H-2K^b^:Ig dimer molecule by incubating at 4°C for 20 min.

### In Vitro Stimulation of Murine Lymphocytes

Splenocytes and IHLs were stimulated with HBV peptide pools, at a concentration of 2 g/mL, for 12 h in the presence of brefeldin A (Sigma) and anti-CD107a. 15-mer synthetic peptides, overlapping by 11 amino acids, spanning the HBV genotype C core, polymerase, envelope and Ad5 hexon proteins were obtained from Mimotopes, Australia. Peptides were reconstituted in DMSO (Sigma-Aldrich), pooled as per requirements and stored at 80°C.

### RT–qPCR

Total RNA was extracted from frozen livers using RNeasy Mini Kit (QIAGEN), according to the manufacturer’s instructions. Genomic DNA contamination was removed using RNAse free DNAse (QIAGEN). 1 μg of total RNA was reverse transcribed using High-Capacity cDNA Reverse Transcription Kit (Applied Biosystems). For viraemia quantification, a standard curve was generated using plasmid DNA containing the HBV genome prepared at 10-fold dilutions (1.33×10^2^-1.33×10^9^ copies/ml). The PCR primer pairs for HBsAg DNA were 5’-TCCGACTCCTCAAGAGATTC-3’ and 5’-CGTTGTCCGTCAGATACAGCA-3’.

### Detection of serum ALT

Serum alanine aminotransferase (ALT) activity was measured using specific bioreaction strips on a Reflovet Plus reader (Roche Diagnostics, Mannheim, Germany).

### Ethical Approval for human samples

This study was approved by the local ethics boards (Wales Research Ethics Committee 4 and UCL Biobank Ethical Review Committee: Research Ethics Committee reference number 16/WA/0289; Brighton and Sussex: Research Ethics Committee reference number 11/LO/0421) and complies with the declaration of Helsinki. All storage of samples obtained complied with the Human Tissue Act 2004.

### Human PMBCs and intrahepatic lymphocytes preparation

PBMCs were isolated from heparinized blood by density centrifugation using Pancoll (PanBiotech). PBMCs were either used immediately or frozen in FBS (Life Technologies) supplemented with 10% DMSO (Sigma-Aldrich). Resected liver tissue from non-diseased associated tissue of tumor resections were cut into small pieces and incubated at 37°C for 30 minutes in 0.01% collagenase IV (Life Technologies) and 0.001% DNaseI (Sigma-Aldrich), submitted to mechanical disruption using a gentleMACS (Miltenyi Biotec) and debris removed by filtration through 70 μm cell strainers (Greiner). Perfusion liquid (perfusate) from healthy livers used for solid-organ transplantation was first concentrated by centrifugation. Parenchymal cells were removed by centrifugation (400 g) on a 30% Percoll gradient (GE Healthcare) and IHLs then isolated by density centrifugation (400 g) using Pancoll (PanBiotech).

### Induction of PD-L1 expression on human NK cells

Human freshly purified NK cells from PBMCs of CHB patients were isolated (>96% purity and viability; NK isolation kit; Miltenyi Biotec) as per manufacturer’s instructions. NK cells were stimulated for 24h with human IL-2 (200U/mL), IL-12 (50ng/mL), IL-15 (50ng/mL) and IL-18 (50ng/mL) (Peprotech). In selected experiments, cells were centrifuged and supernatant was harvested. Cells were stained with CFSE (Invitrogen) as per manufacturer’s instructions and treated with anti-PD-L1 (10μg/mL) (12-5983-42, eBioscience) or isotype control for 1h. In some experiments, cells were also treated with 10μg/mL of anti-NKG2D (1D11, eBioscience) or TRAIL-R2-Fc (R&D Systems). Cells were washed 3 times transferred to 96-well plates containing peptide-expanded PBMCs.

### Short-term culture of human PBMCs

PBMCs depleted of NK cells (3×10^5^ cells/well) were stimulated with 1 mM of a pool of 15-mer peptides overlapping by 10 residues spanning the core protein of HBV genotype D (JPT Peptide Techonologies) in the presence of 20 IU/mL IL-2 in RPMI complete medium for 7 days at 37°C. IL-2 and medium were refreshed on day 4 of culture. On day 7, PBMCs were re-stimulated with 1 mM of peptide pool, Brefeldin A (1μg/mL) was added 2h later and cells were incubated overnight. Where applicable, a physiological ratio (based on the patient-specific circulating NK cell frequency) of cytokine treated NK cells was re-added in the culture at the onset of re-stimulation. In selected experiments, 10μg/mL of anti-IFNγ, was added during overnight peptide re-stimulation (NIB42, eBioscience). For some experiments, 100µl of supernatant harvested after NK cell stimulation was added for the peptide overnight re-stimulation and media containing the cytokine cocktail was used for the peptide only wells. Where indicated, separated cytokine-activated NK cells were plated into transwells (1µm pore size; Polycarbonated Membrane; Corning Costar).

### Flow cytometry

Multiparametric flow cytometry was used for phenotypic and functional analysis of mouse splenocytes and IHLs and human PBMCs and IHLs (antibodies are listed in Suppl. Table 1). Cells were stained with a fixable Live/Dead dye (Invitrogen) before incubation with saturating concentrations of surface mAbs diluted in 50% Brilliant violet buffer (BD) and 50% PBS for 30 min at 4°C. Cells were fixed and permeabilized for further functional assessment with either Cytofix/Cytoperm or Cytofix (BD) according to the manufacturer’s instructions. For intracellular cytokine staining (ICS), samples were then incubated with saturated concentrations of intracellular mAbs for 30 min at 4°C diluted in 0.1% saponin (Sigma-Aldrich). All samples were acquired on BD Fortessa X20 flow cytometer (BD) and analyzed using FlowJo (Tree Star).

### Statistics

Statistical analyses were performed in Prism (GraphPad) as indicated in legends: Kruskal-Wallis test (ANOVA) with Dunn’s post hoc test for pairwise multiple comparisons; Mann-Whitney unpaired t test; and Wilcoxon’s paired t test. Significance was defined as P < 0.05.

## Supplementary Material

Reproducibility Checklist

Supplementary Data

## Data Availability

All data associated with this study are available in the main text or the supplementary materials

## Single sentence summary

Manipulation of NK cell calibration of T cells can enhance the response to therapeutic adenoviral vector vaccination in chronic HBV infection
